# The Effects of Heparan Sulfate Infusion on Endothelial and Organ Injury in a Rat Pneumosepsis Model

**DOI:** 10.3390/jcm12206438

**Published:** 2023-10-10

**Authors:** Daan P. van den Brink, Derek J. B. Kleinveld, Annabel Bongers, Jaël Vos, Joris J. T. H. Roelofs, Nina C. Weber, Jaap D. van Buul, Nicole P. Juffermans

**Affiliations:** 1 Amsterdam UMC, Department of Intensive Care Medicine, University of Amsterdam, 1105 AZ Amsterdam, The Netherlands; 2 Amsterdam UMC, Laboratory of Experimental Intensive Care and Anesthesiology, University of Amsterdam, 1105 AZ Amsterdam, The Netherlands; d.j.kleinveld@amsterdamumc.nl (D.J.B.K.); a.bongers@hubrecht.nl (A.B.); j.vos1@amsterdamumc.nl (J.V.); n.c.hauck@amsterdamumc.nl (N.C.W.); n.juffermans@erasmusmc.nl (N.P.J.); 3 Erasmus MC, Department Anesthesiology, Erasmus University of Rotterdam, 3015 GD Rotterdam, The Netherlands; 4 Amsterdam UMC, Department of Pathology, University of Amsterdam, 1105 AZ Amsterdam, The Netherlands; j.j.roelofs@amsterdamumc.nl; 5 Amsterdam UMC, Cardiovascular Sciences, 1105 AZ Amsterdam, The Netherlands; 6 Sanquin Research and Landsteiner Laboratory, Molecular Cell Biology Laboratory, Department Molecular Hematology, 1066 CX Amsterdam, The Netherlands; j.vanbuul@sanquin.nl; 7 Leeuwenhoek Centre for Advanced Microscopy (LCAM), Section Molecular Cytology at Swammerdam Institute for Life Sciences (SILS), University of Amsterdam, 1066 CX Amsterdam, The Netherlands; 8 Erasmus MC, Department of Intensive Care, Erasmus University of Rotterdam, 3015 GD Rotterdam, The Netherlands

**Keywords:** endothelium, resuscitation, sepsis, shock, heparan sulfate, animal model

## Abstract

Septic shock is characterized by endothelial dysfunction, leading to tissue edema and organ failure. Heparan sulfate (HS) is essential for vascular barrier integrity, possibly via albumin as a carrier. We hypothesized that supplementing fluid resuscitation with HS would improve endothelial barrier function, thereby reducing organ edema and injury in a rat pneumosepsis model. Following intratracheal inoculation with Streptococcus pneumoniae, Sprague Dawley rats were randomized to resuscitation with a fixed volume of either Ringer’s Lactate (RL, standard of care), RL supplemented with 7 mg/kg HS, 5% human albumin, or 5% human albumin supplemented with 7 mg/kg HS (*n* = 11 per group). Controls were sham inoculated animals. Five hours after the start of resuscitation, animals were sacrificed. To assess endothelial permeability, 70 kD FITC-labelled dextran was administered before sacrifice. Blood samples were taken to assess markers of endothelial and organ injury. Organs were harvested to quantify pulmonary FITC-dextran leakage, organ edema, and for histology. Inoculation resulted in sepsis, with increased lactate levels, pulmonary FITC-dextran leakage, pulmonary edema, and pulmonary histologic injury scores compared to healthy controls. RL supplemented with HS did not reduce median pulmonary FITC-dextran leakage compared to RL alone (95.1 CI [62.0–105.3] vs. 87.1 CI [68.9–139.3] µg/mL, *p* = 0.76). Similarly, albumin supplemented with HS did not reduce pulmonary FITC-dextran leakage compared to albumin (120.0 [93.8–141.2] vs. 116.2 [61.7 vs. 160.8] µg/mL, *p* = 0.86). No differences were found in organ injury between groups. Heparan sulfate, as an add-on therapy to RL or albumin resuscitation, did not reduce organ or endothelial injury in a rat pneumosepsis model. Higher doses of heparan sulfate may decrease organ and endothelial injury induced by shock.

## 1. Introduction

Sepsis is a major cause of mortality and is characterized by inflammation-induced endothelial dysfunction [1,2]. Under healthy conditions, the inner cell wall of endothelial cells is lined with the glycocalyx [3]; this dynamic structure is involved in numerous microvascular processes, including vascular permeability, hemostasis, and leukocyte adhesion [4]. During sepsis, a disturbed inflammatory host response leads to the degradation of the endothelial glycocalyx [2,5,6], exposing the procoagulant endothelial cell surface and reducing endothelial barrier function. This results in microthrombi formation, endothelial permeability, organ edema, and disturbed tissue perfusion, leading to organ damage and poor outcomes [2]; therefore, treatments aiming to reduce endothelial injury may be beneficial in sepsis [7].

Heparan sulfate (HS) is one of the major constituents of the endothelial glycocalyx [8]. In sepsis, levels of circulating HS are increased; higher circulating HS levels are associated with poor outcome and is hence used as a biomarker for glycocalyx degradation and sepsis severity [9,10]. HS is a linear polysaccharide that possesses multiple negatively charged highly sulfated regions, allowing it to interact with numerous circulating, positively charged, heparan sulfate binding proteins (HSBP), including growth factors, antimicrobial peptides, and adhesion molecules [11]. Through these interactions, HS could protect the endothelial barrier. For instance, HS promotes reconstitution of the endothelial glycocalyx by regulating the activation of fibroblast growth factor 2 (FGF2)—FGF receptor 1 (FGFR1) signaling—which is suppressed during sepsis [12] and via direct incorporation of HS into the glycocalyx [13]. Moreover, HS has anticoagulant properties by activating antithrombin III and by inhibiting coagulation factors Xa and IIa [14,15], via which HS could reduce microthrombi formation. In a mouse model of histone-induced lung injury, the supplementation of HS protected endothelial membrane integrity and attenuated acute lung injury [16]. Therefore, HS, in addition to fluid resuscitation, may be beneficial.

The aim of this study was to investigate the effects of HS as an add-on therapy to resuscitation with Ringer’s lactate (RL). As albumin may be a carrier protein for HS, additional groups receiving albumin and HS were added. We hypothesized that HS would reduce endothelial and glycocalyx injury, thereby limiting organ edema and injury.

## 2. Materials and Methods

### 2.1. General Information

This study was approved by the Animal Care and Use Committee of the Amsterdam University Medical Centers, location AMC, at the University of Amsterdam, the Netherlands. The procedures were performed in accordance with the European Parliament directive (2010/63/EU) and the national law of the Experiments on Animals Act (Wod, 2014). Male Sprague Dawley rats (Envigo, Indianapolis, IN, USA), 12–13 weeks old, weighing approximately 400 g, were group-housed in standard cages under normal conditions of a 12:12-h light:dark cycle with access to food and water ad libitum for ≥7 days before the experiment. Studies were conducted and reported following the ARRIVE guidelines (Supplementary Material) [17].

### 2.2. Pneumosepsis Model

*Streptococcus pneumoniae* (serotype 3, ATCC 6303; Rockville, MD, USA) were cultured on Colombian agar with 5% sheep blood plates (Biomerieux Benelux B.V., Zaltbommel, The Netherlands). Cultures were transferred to brain heart infusion broth and incubated overnight at 37 °C. Thereafter, cultures were centrifuged twice at 2700× *g*, 20 °C, acceleration 9, deceleration 3 (5804R, Eppendorf AG, Hamburg, Germany), and resuspended in 0.9% NaCl to create the inoculum. Assuming that an OD_620_ of 0.350 = 1 × 10^8^ colony forming units (CFU), inocula were prepared using a spectrophotometer (Secoman S250). Inocula were plated in serial 10-fold dilutions and grown overnight for quantification.

Rats were intratracheally inoculated with 3–5 × 10^8^ CFU *S. pneumoniae* in 100 µL sterile 0.9% NaCl while anesthetized with 5% isoflurane in 100% FiO_2_. During pilot experiments, this amount induced pneumosepsis in 100% of animals, defined as lactatemia, with the growth of *S. pneumoniae* in the lung and blood cultures after 29 h. The following exclusion criteria were established before the start of experiments: animals receiving too low (<3 × 10^8^ CFU) or too high (>5 × 10^8^ CFU) amounts of *S. pneumoniae*; animals without growth of *S. pneumoniae* in either the lung or blood cultures; animals that died before the start of the intervention.

**Figure A1:**
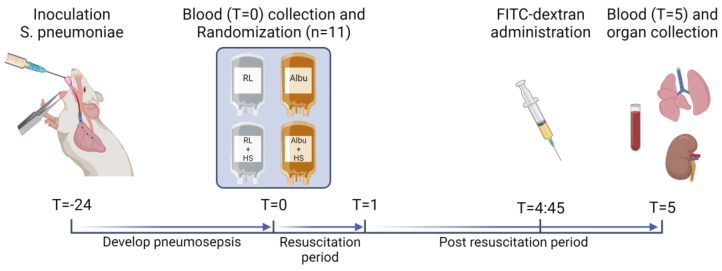
Schematic overview of experimental model.

Twenty-four hours after inoculation (T = 0) (Figure A1, Appendix A), animals were anesthetized with a 1–3% isoflurane/60% FiO_2_ mix, whereafter a 21G Venflon was introduced into the tail vein to administer interventions. Before receiving anesthetics, animals were randomized (*n* = 11 per group) using opaque envelopes in a 1:1 allocation to receive a fixed volume of 8 mL/kg of either Ringer’s lactate (RL), Ringer’s lactate + 7 mg/kg heparan sulfate (RL + HS) (MedChem Express, Monmouth Junction, NJ, USA), 5% human albumin (albu), or 5% human albumin + 7 mg/kg heparan sulfate (albu + HS) via a perfusion pump (^®^ fm; B Braun, West Bloomfield Township, MI, USA). The used dose of heparan sulfate was based on literature [18]. Healthy controls (*n* = 6) underwent all procedures but were sham inoculated with 100 µL sterile NaCl 0.9% (sham). Both control groups were randomized together with the intervention groups. Researchers were not blinded to group allocation during the conduct of the experiment and data analyses. 

After the resuscitation period, the Venflon was removed and anesthesia was stopped. Five hours after the start of resuscitation, rats were anesthetized using 1–3% isoflurane and exsanguinated through heart puncture (T = 5). Fifteen minutes before sacrifice, 6.25 mg 70 kDa fluorescein isothiocyanate (FITC)-labeled dextran (Sigma Aldrich, St. Louis, MO, USA) in 0.5 mL saline was administered. Immediately after exsanguination, 2 mL of heparinized saline (1250 iE/mL, Leo Pharma BV, Amsterdam, The Netherlands) was administered intraventricular to prevent clotting. Thoracotomy and laparotomy were performed, and the vena cava inferior was cut. Using 100 mL 0.9% NaCl, the circulation was flushed in order to remove excess intravascular FITC-dextran. Before flushing, the left pulmonary and renal hilum were ligated to prevent flushing in order to assess them for wet-to-dry (W/D) ratios. After flushing, both the lungs and kidneys were removed for later assessment. Urine was collected by bladder puncture.

### 2.3. Dose–Response Study

**Figure A2:**
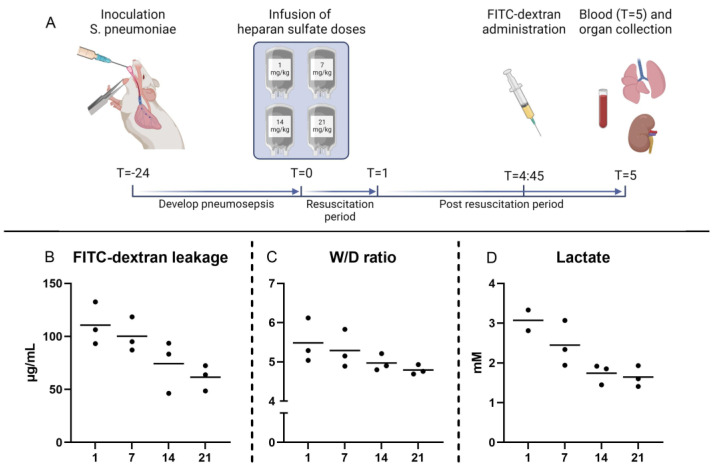
Dose-response study heparan sulfate. (**A**) Overview of dose-response study. Four doses of heparan sulfate were tested: 1 mg/kg, 7 mg/kg, 14 mg/kg and 21 mg/kg. Heparan sulfate was infused in 8 mL/kg Ringer’s Lactate. 3 animals per group were included. (**B**–**D**) data points are shown with mean. Pulmonary FITC-dextran leakage and W/D ratios are shown, lactate was measured at T = 5. FITC = Fluorescein isothiocyanate, W/D ratio = Wet-to-dry ratio.

A dose–response study was conducted to investigate the effects of heparan sulfate at four doses: 1 mg/kg, 7 mg/kg, 14 mg/kg, and 21 mg/kg (Figure A2, Appendix A). Heparan sulfate was infused in 8 mL/kg Ringer’s lactate, with 3 animals assigned to each group. The dose–response study was carried out after the experiment to determine whether the potential beneficial effects of heparan sulfate at higher or lower doses were missed. The range of the used doses was based on previous literature [19].

### 2.4. Animal Welfare

Using a modified M-CASS scoring sheet (Table A2, Appendix A) [20,21], animals were monitored during the whole study period using predefined moments by the responsible researcher (after inoculation, T = 8, T = 24, T = 26.5, and T = 29) or animal caretaker (T = 8, T = 20). Scoring 4 points in any of the categories would result in early termination. When observing the unusual progression of symptoms, monitoring frequency was increased. An autopsy was performed on all animals not reaching the end of the study period to assess the cause of death. 

### 2.5. Measurements

Blood samples were drawn pre-resuscitation (T = 0) and at sacrifice (T = 5). Blood gas analyses (RAPIDPoint 500, Siemens, Munich, Germany) and total blood counts (cell Coulter AC•T diff2 Hematology analyzer, Beckmann, Germany) were undertaken. After collection, blood was centrifuged twice (2000× *g*, 15 min, acceleration 9, brake 0, 18 °C; 5804 R, Eppendorf AG, Hamburg, Germany), aliquoted, snap frozen in liquid nitrogen, and stored at −80 °C until further evaluation. Using commercially available ELISA kits, levels of syndecan-1 (Elabscience, Houston, TX, USA), thrombomodulin (TM) (Lifespan Biosciences, Inc., Linwood, DC, USA), von Willebrand factor (vWF) (Elabscience, Houston, TX, USA), intercellular adhesion molecule-1 (ICAM-1) (Elabscience, Houston, TX, USA), IL-6 (R&D, Minneapolis, MI, USA), and TNF-a (R&D, Minneapolis, MI, USA) were determined according to manufacturer guidelines. Aspartate transaminase (AST), alanine transaminase (ALT), albumin, and creatinine were measured by the Amsterdam UMC, AMC Laboratory of Clinical Chemistry (LAKC) by standard enzymatic reactions using spectrophotometric, colorimetric, or turbidimetric measurement methods. 

### 2.6. Pulmonary Vascular Leakage

To measure the amount of FITC-dextran that had leaked into the lung, lung tissue was thawed and homogenized using a radioimmunoprecipitation assay (RIPA) buffer and tissue homogenizer (TH-115, Omni International, Kennesaw, GA, USA). A 100 µL sample of this solution was measured for fluorescence using a spectrophotometer (spectramax M2, molecular devices, San Jose, CA, USA). 

### 2.7. Histopathology

The lower lobe of the right lung was paraffinized, sliced in 4-µm-thick slices, and stained with hematoxylin and eosin (H&E). Thereafter, lung histology was examined by a pathologist blinded for treatment allocation. The lungs were scored on the presence of edema and inflammation on a scale of 0–4 (0 = absent, 4 = very severe). For the full scoring list, see the Appendix A (Table A1). Representative sections were selected with microscopy (LEICA DM 2000, Leica Microsystems, Wetzlar, Germany).

### 2.8. Bacterial Growth

The lower lobe of the right lung was homogenized in sterile 0.9% NaCL using a tissue homogenizer (TH-115, Omni international, Kennesaw, GA, USA). Lung homogenates were plated in serial 10-fold dilutions. Whole blood retrieved during termination (T = 5) was plated, and the growth of *S. pneumonia* in the whole blood was defined as bacteremia.

### 2.9. Sample Size and Statistical Analyses

The primary outcome in the current model is pulmonary FITC-dextran leakage. The previous literature, using similar models, did not measure pulmonary FITC-dextran leakage but did measure syndecan-1 levels. Pulmonary FITC-dextran leakage serves as a marker of endothelial permeability, and syndecan-1 is a marker of glycocalyx degradation. As both measurements are closely related, we expected comparable increases in the current model; therefore, our sample size calculation was based on a previous sepsis model, comparing resuscitation with plasma to normal saline, which reduced syndecan-1 levels [21]. 

These data were extrapolated to a sample size of 7 groups. Using a one-way ANOVA analysis of variance (V = 17.5) and a common standard deviation of 9, the use of 10 rats had a power of 80% to reach a statistically significant difference in vascular leakage. With an expected mortality of 5% in our model, 11 rats were included per group.

Statistical analysis was carried out using SPSS Statistics V.26 (IBM). Graphs were made using GraphPad Prism 8 (GraphPad Software). All data were regarded as non-parametric and are shown as median with interquartile range (IQR). Data were analyzed using the Kruskal–Wallis test with post hoc multiple comparisons; no correction for multiple testing was performed as data was considered explorative. A *p*-value < 0.05 was considered statistically significant. Data collected until the moment of death was used in analyses. Measurement values of used techniques that were lower than the reference value were set at the lowest detection range.

## 3. Results

### 3.1. Pneumosepsis Model

**Figure 1:**
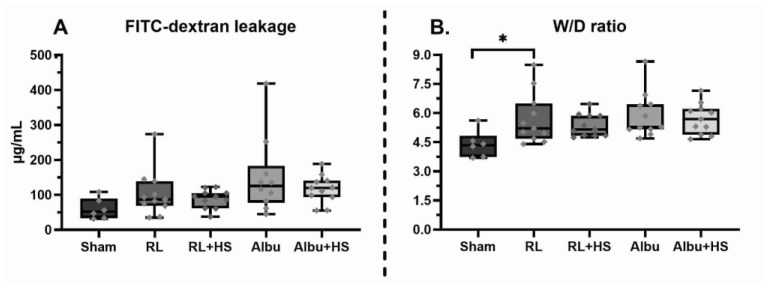
Effect of resuscitation with and without HS on markers of pulmonary injury. Data are presented as boxplots showing all data points., Albu = 5% human albumin, FITC = fluorescein isothiocyanate, HS = 7 mg/kg heparan sulfate, RL = Ringer’s lactate, W/D ratio = wet-to-dry ratio. * *p* < 0.05.

**Figure 2:**
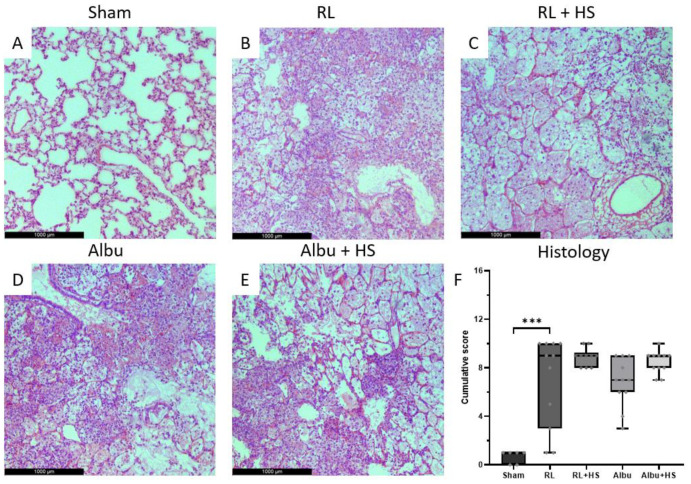
Effect of resuscitation with and without HS on pulmonary histologic injury. (**A**–**E**) H&E staining of representative lung tissue in sham, RL, RL + HS, albu, and albu + HS groups. (**F**) Histological score of lung tissue in each group. Data are presented as boxplots showing all data points. Albu = 5% human albumin, HS = 7 mg/kg heparan sulfate, RL = Ringer’s lactate. *** *p* < 0.001.

All animals inoculated with *S. pneumoniae* developed pneumosepsis with lactatemia (median: 1.56 [IQR: 0.87–1.69] vs. 2.24 [2.10–2.71] mM, *p* < 0.05) and growth in both lung homogenates and blood cultures compared to sham. Septic animals had increased weight loss compared to sham animals (median: 8.1 [IQR: 7.1–9.1] vs. 0.9 [0.3–1.4]%) and elevated potassium and base excess levels while leucocyte, platelet, and calcium levels were decreased (Table 1). No baseline differences were seen between groups prior to intervention (Table 1). Sepsis led to pulmonary injury, as evidenced by increased histopathology scores (*p* < 0.05) and lung W/D ratios (*p* ≤ 0.01). Also, there were increased levels of soluble TM (*p* < 0.01) and syndecan-1 (*p* < 0.01) compared to sham animals (Figure 1 and Figure 2). Hepatic or renal injury biomarkers were comparable between septic animals (Table 2). One animal was terminated early upon reaching humane endpoints (albumin: 1, Results A1, Appendix A).

### 3.2. Effects of Heparan Sulfate on Pulmonary Injury

RL supplemented with HS resuscitation did not reduce pulmonary FITC-dextran leakage (95.1 [62.0–105.3]) compared to RL resuscitation (87.1 [68.9–139.3] µg/mL, *p* = 0.76) (Figure 1). Moreover, no differences were seen in pulmonary W/D ratios (*p* = 0.95, Figure 1), pulmonary histology scores (*p* = 0.28, Figure 2), and lung homogenate bacterial outgrowth (*p* = 0.20, Table 2). Similarly, albumin supplemented with HS resuscitation did not show a decrease in pulmonary FITC-dextran leakage (120.0 [93.8–141.2] vs. 116.2 [61.7–160.8] µg/mL, *p* = 0.86), W/D ratios (*p* = 0.99), histology (*p* = 0.12), and bacterial outgrowth (*p* = 0.37) when compared to albumin resuscitation (Figure 1 and Figure A1, Appendix A). Of interest, in the dose–response study, animals administered the highest dose of HS exhibited the lowest pulmonary FITC-dextran leakage (mean = 61.6 µg/mL) and pulmonary W/D ratios (4.79), whereas animals receiving the lowest dose of HS had the highest pulmonary FITC-dextran leakage (110.7) and pulmonary W/D ratios (5.48) (Figure A2).

### 3.3. Effects of Heparan Sulfate on Systemic Injury

**Figure 3:**
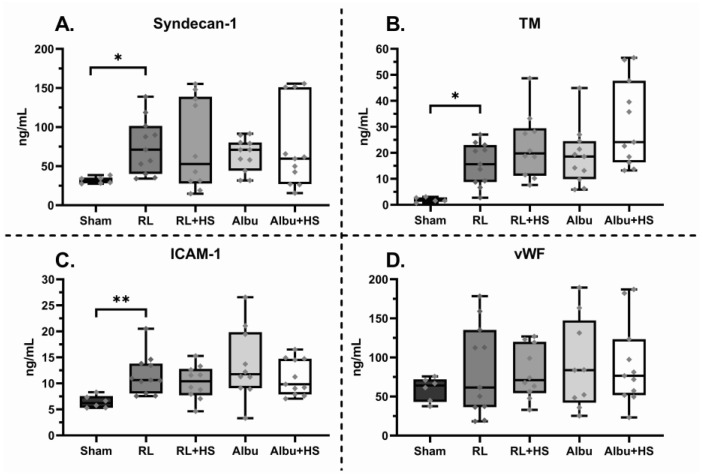
Effect of resuscitation with and without HS on markers of markers of endothelial injury, activation, and inflammation. Data are presented as boxplots showing all data points. Albu= 5% human albumin, HS = 7 mg/kg heparan sulfate, ICAM-1 = intercellular adhesion molecule 1, RL = Ringer’s lactate, TM = thrombomodulin, vWF = von Willebrand factor. * *p* < 0.05, ** *p* < 0.01.

Resuscitation significantly increased kidney W/D ratios compared to sham animals (Table 2). The addition of HS to resuscitation with RL or albumin did not improve markers of shock and organ injury post-resuscitation (Table 2). Moreover, all groups had comparable levels of syndecan-1, sTM, vWF, and ICAM-1 (Figure 3). Regarding inflammation, septic animals had increased levels of IL-6 and TNF-α in lung homogenates and increased levels of IL-6 in plasma (Table 2). However, no differences in inflammation were seen between animals receiving HS and animals not receiving HS.

## 4. Discussion

HS as an add-on therapy to resuscitation with either Ringer’s lactate or albumin did not reduce endothelial and organ injury and inflammation when compared to resuscitation without HS. These data suggest that 7 mg/kg HS infusion does not prevent glycocalyx degradation and endothelial injury in pneumosepsis. 

An explanation for the absence of protective effects of HS could be the fact that HS is able to bind numerous different mediators [22]. HS are linear polysaccharides with very high structural variability. Due to structural modification reactions after synthesis, HS differs in saccharide chain length, epimerization, and sulfation patterns. These differences dictate HS-protein binding affinity and selectivity [23]. Likely, HS can be beneficial through the binding of certain mediators, such as fibroblast growth factor (FGF) and histones, promoting reconstitution of the glycocalyx and reducing endothelial injury [4,24]. However, HS also binds mediators, activating harmful pathways, neutralizing key components in host defense, or disrupting growth factors necessary for vascular homeostasis [25]. Also, HS may act as a damage-associated molecular pattern, exacerbating the inflammatory response [26]. However, as cytokine levels did not differ between HS and control, this explanation appears less likely. The net effect of the positive and negative effects could be neutral, resulting in no reduction of endothelial injury. Also, the effects of HS depend on the HS blood concentration. While low concentrations of HS have been found to activate FGF2 by facilitating growth factor ligand–receptor complex formation [27], high concentrations of HS compete to bind both the ligand and receptor, preventing the complex formation and inhibiting downstream signaling [28]; thereby, counteracting severe shedding of HS in sepsis with exogenous HS may potentially inhibit rather than activate endothelial reparative pathways.

HS promotes anticoagulation by activating antithrombin III and inhibiting factor Xa and IXa, thereby possibly preventing thrombi formation [14,15]. In the current model, TM levels were elevated and platelet levels were decreased compared to the sham group, indicative of activation of the coagulation system. However, supplementation of HS did not seem to inhibit this effect. Importantly, in lung histology, no thrombi formation was observed in animals receiving and not receiving HS. Possibly, the used model did not result in microthrombi formation.

Our findings, in which HS did not limit endothelial or organ injury when added to Ringer’s lactate or albumin resuscitation, were in contrast to a previous study in a histone-induced acute lung injury mouse model, wherein HS attenuated lung injury [16]. An explanation for these disparate results may be the use of a bacterial infection sepsis model, which more closely resembles clinical sepsis and is not restricted to histone-induced injury. Alternatively, the HS dose may have been too low. The used dose was based on a previous study in a shock model [18]. After conducting the experiments in this manuscript, a dose–response study was conducted to investigate this. The group receiving the highest dose of 21 mg/kg HS had the least amount of pulmonary FITC-dextran leakage and pulmonary edema (Figure A2). This finding corresponds with a recent study that showed that 20 mg/kg HS improved survival and reduced inflammation in a mice CLP model [19]. However, due to the low sample size, this may be a chance finding and should be confirmed in follow up studies. Also, the volume of resuscitation fluid may have been too modest. After resuscitation, lactate levels did not normalize, indicating perhaps only limited volume loading. In septic patients, the amount of volume is associated with increased glycocalyx shedding [29]. The used dose of 8 mL/kg translates to approximately 600 mL of administered fluids. A recent RCT comparing restrictive vs. liberal resuscitation for septic shock patients showed that a median difference of 700 mL did not influence outcomes. Possibly, the beneficial effects of HS supplementation may be apparent only after high-volume resuscitation [30].

This study has several limitations. Firstly, we witnessed higher data variability than initially expected. Therefore, the used sample size may have been too small to detect potential differences between interventions. Also, control groups were added to define the sepsis model, but without correcting for multiple testing, which increases the probability of type 1 errors. The HS dosage was based on a traumatic shock model; therefore, the used dosage could have been too high or too low in sepsis. The dose–response study was carried out after conducting the experiments showing that a too-low dose was likely used. Also, rats were followed for 5 h after the start of resuscitation to see the short-term effects of HS on endothelial injury. However, long-term effects on endothelial and organ injury were not evaluated.

## 5. Conclusions

The addition of HS to resuscitation with either Ringer’s lactate or albumin did not reduce endothelial or organ injury in a rat pneumosepsis model. Higher doses of heparan sulfate may decrease organ and endothelial injury induced by shock.

## Figures and Tables

**Table 1:** Pre-resuscitation parameters (T = 0).

Parameter	Sham (*n* = 6)	RL (*n* = 11)	RL + HS (*n* = 11)	Albu (*n* = 11)	Albu + HS (*n* = 11)
Weight loss (%)	0.9 (0.3–1.4)	7.5 * (6.9–9.5)	8.2 * (7.3–8.7)	8.2 * (7.4–9.3)	7.8 * (7.3–8.5)
Vitals					
Heart rate (bpm)	370 (344–385)	367 (340–377)	340 (314–360)	365 (338–381)	364 (324–379)
Saturation (%)	97 (95–99)	98 (97–99)	96 (94–98)	95 (93–98)	97 (93–98)
Blood count					
Hb (mM)	10.1 (10.0–11.1)	10.6 (10.0–12.0)	11.5 (10.6–11.9)	11.1 (10.8–11.7)	11.7 (10.2–12.2)
Leukocytes (*10^9^/L)	12.0 (7.1–14.6)	2.6 * (1.1–8.6)	2.7 (1.6–4.5)	4.1 * (1.5–9.1)	1.9 * (1.3–2.9)
Platelets (*10^9^/L)	965 (743–989)	678 * (452–865)	621 * (353–812)	775 (580–917)	668 * (546–849)
Blood gas					
Lactate (mM)	1.98 (1.62–2.05)	2.55 * (2.37–2.72)	2.77 * (2.36–2.83)	2.51 * (2.41–3.04)	2.71 * (2.50–2.79)
pH	7.42 (7.41–7.44)	7.43 (7.42–7.45)	7.44 (7.39–7.45)	7.41 (7.38–7.44)	7.44 (7.40–7.46)
BE (mM)	3.2 (2.1–4.6)	4.6 (3.6–5.9)	6.0 * (5.0–6.6)	4.6 (2.6–6.9)	5.3 * (4.2–6.1)
HCO_3_^−^ (mM)	28.2 (27.2–29.8)	30.2 * (28.0–32.0)	31.8 * (30.2–33.5)	31.0 * (28.6–32.2)	30.7 * (29.9–32.4)
Natrium (mM)	138 (137–139)	139 (137–139)	139 (138–139)	139 (138–140)	139 (137–140)
Potassium (mM)	5.3 (5.1–5.4)	5.9 * (5.3–6.6)	5.9 * (5.3–6.1)	5.6 * (5.4–5.8)	5.7 * (5.4–5.9)
Calcium (mM)	1.21 (1.19–1.25)	1.12 * (1.04–1.17)	1.12 * (1.08–1.15)	1.16 * (1.09–1.19)	1.09 * (1.07–1.16)
Glucose (mM)	8.9 (7.5–12.2)	8.0 (7.4–8.9)	7.2 (6.9–8.5)	7.6 (7.4–9.5)	7.2 (6.7–7.7)

Data are presented as median (inter-quartile range). * *p* < 0.05 when compared to the sham group. Albu = albumin, BE = base excess, bpm = beats per minute, Hb = hemoglobin, HS = heparan sulfate, RL = Ringer’s lactate.

**Table 2:** Post-resuscitation parameters (T = 5).

Parameter	Sham (*n* = 6)	RL (*n* = 11)	RL + HS (*n* = 11)	Albu (*n* = 10)	Albu + HS (*n* = 11)
Blood gas					
Lactate (mM)	1.56 (0.87–1.69)	2.24 * (2.10–2.71)	2.18 * (2.00–2.89)	2.12 * (2.04–2.67)	2.74 * (2.08–2.99)
Hb (mM)	8.9 (8.3–9.4)	10.1 * (9.4–11.6)	10.9 * (10.4–12.5)	10.4 * (9.6–10.9)	11.7 * (10.3–12.7)
Glucose (mM)	10.3 (9.5–11.6)	8.2 * (7.1–9.4)	6.9 * (6.5–7.4)	7.9 * (7.6–8.1)	7.6 * (6.6–8.3)
HCO_3_^−^ (mM)	29.9 (27.5–32.8)	29.4 (26.6–30.5)	30.6 (28.4–31.1)	30.8 (30–32.6)	28.7 (25.6–30.2)
Saturation (%)	98 (98–99)	94 (90–96)	96 (94–97)	96 (92–96)	94 (93–97)
Organ injury					
ALT (U/L)	34.0 (30.8–38.3)	35.0 (30.0–38.3)	32.0 (25.8–39)	34.5 (29.8–37.3)	32.5 (25.8–35.3)
AST (U/L)	64.5 (59.8–73.5)	75.0 (54.0–82.5)	92.0 (61.8–101.5)	86.0 (62.0–95.5)	66.0 (58.5–99.0)
Albumin (g/L)	36.5 (33.5–39.0)	36.0 (34.0–38.0)	35 (33.5–37.3)	37 (35.5–38.3)	35 (32.8–37.3)
Creatinine (µM)	23.5 (19.5–28.3)	23.0 (21.8–25)	22 (20–25.5)	21.0 (20.0–25.0)	23.0 (22.0–24.8)
Kidney W/D ratio	3.6 (3.4–4.0)	3.9 * (3.7–4.1)	3.9 * (3.8–4.0)	4.0 * (3.7–4.1)	3.9 * (3.9–4.1)
Inflammation					
IL-6 plasma	12.4 (12.4–12.4)	262.3 * (67.6–1119.9)	349.7 * (142.0–619.4)	567.4 * (329.5–1311.5)	745.7 * (117.4–971.6)
IL-6 lung homogenate (ng/mL)	18.0 (16.1–19.0)	21.9 (18.7–35.4)	36.1 * (31.8–47.1)	41.6 * (24.1–46.0)	41.6 * (30.5–45.1)
TNF-α lung homogenate (ng/mL)	5.0 (4.8–6.4)	10.6 (5.4–15.2)	14.4 * (12.6–23.6)	18.0 * (11.9–28.8)	12.4 * (11.0–25.7)
Lung homogenate bacterial outgrowth (CFU/gram)	0 * (0–0)	6.0 × 10^7^ * (1.0 × 10^7^–1.3 × 10^8^)	6.0 × 10^7^ * (3.4 × 10^7^–1.6 × 10^8^)	7.0 × 10^7^ * (1.3 × 10^7^–3.6 × 10^8^)	8.1 × 10^7^ * (6 × 10^7^–1.6 × 10^8^)

Data are presented as median (inter-quartile range). * *p* < 0.05 when compared to the sham group. Albu = 5% human albumin, ALT = alanine aminotransferase, AST = aspartate aminotransferase, bpm = beats per minute, CFU = colony forming units, Hb = hemoglobin, HS = heparan sulfate, IL-6 = interleukin 6, RL = Ringer’s lactate, TNF-α = tumor necrosis factor-alpha, W/D ratio = wet-to-dry ratio.

## Data Availability

Data are available upon request due to restrictions, e.g., privacy or ethics. The data presented in this study are available on request from the corresponding author.

## Supplementary Materials

The following supporting information can be downloaded at: https://www.mdpi.com/2077-0383/12/20/6438/s1.

## Appendix A

**Table A1:** Lung injury assessment scoring list.

Group	Sham (n = 6)	RL (n = 11)	RL + HS (n = 11)	Albu (n = 10)	Albu + HS (n = 11)
Interstitial inflammation	0 (0–1)	2 * (1–2)	2 * (1–3)	2 (1–2)	1 * (0–2)
Endothelialitis	0 (0–0)	1 * (0–2)	0 (0–0)	0 (0–0)	0 (0–0)
Bronchitis	0 (0–0)	3 * (1–3)	3 * (3–4)	3 * (2–3)	4 * (3–4)
Edema	0 (0–1)	3 * (1–3)	3 * (3–4)	3 * (3–3)	4 * (3–4)
Thrombi formation	0 (0–0)	0 (0–0)	0 (0–0)	0 (0–0)	0 (0–0)

Data are presented as median (inter-quartile range). * *p* < 0.05 when compared to sham group. Severity was assessed based on a scale from 0–4 (0, absent; 1, mild; 2, moderate; 3, severe; 4 = very severe). * *p* < 0.05 when compared to the sham group, Albu = human albumine 5%, HS = Heparan sulfate, RL = Ringer’s lactate.

Description of pneumosepsis rat model. Animals were intratracheally inoculated 3–5 × 10^8^ *Streptococcus pneumoniae* whereafter they developed pneumosepsis. 24 h after inoculation, animals were anesthetized using isoflurane and blood was collected (T = 0). Thereafter, animals were randomized to resuscitation with Ringer’s lactate (RL), Ringer’s lactate + heparan sulfate (RL + HS), human albumin 5% (albu) or human albumin 5% + heparan sulfate (Albu + HS). Healthy controls were animals receiving sham inoculation. Fluids were administered at 8 mL/kg in 1 h after which resuscitation was stopped and animals were allowed to wake up. The used heparan sulfate dose was 7 mg/kg. Four hours and forty-five minutes after start of transfusion (T = 4:45), animals were anesthetized using isoflurane and 70 kD FITC-labelled-dextran was administered. FITC-dextran circulated for 15 min. Then, blood was collected by heart puncture, quickly followed by a bolus of heparin, tying-off of the hilum of left lung and left kidney (for later W/D ratio assessments). The circulation was flushed using 50 mL of 0.9% NaCl against gravitational force to remove excess FITC-dextrans. Organs were harvested for endothelial leakage assessments and organ failure scores.

Results A1: Mortality and humane endpoints.

1 rat receiving albumin was sacrificed 4 h after receiving intervention upon showing severe dyspnea with abdominal breathing which was defined as a humane end point.

**Table A2:** Pneumosepsis model Monitoring Sheet.

Fur Aspect	Actively Grooming	Dulling of Hair Coat	Rough Hard Coat	Piloerection
Activity	Normal	Reduced activity disturbed	No activity disturbed Reduced activity stimulated	Nil activity disturbed or stimulated
Behavior	Normal, no abd splinting	Slightly hunched, moving freely, mild splinting	Hunched with stiff movement/posture, moderate splinting	Hunched with no movement stimulated, severe splinting
Face	Normal	Normal eyelid opening when disturbed	Orbital tightening, moderate grimacing	Eyelids closed, obvious grimacing
Diarrhea	None	Mild	Moderate	Severe
Respiratory distress	None	Mild dyspnea	Moderate dyspnea	Severe dyspnea with abdominal breathing
Score	1	2	3	4

A score of 4 in any of the categories results in early termination.

Adapted from [20,21].
